# Circulating neurofilament light in ischemic stroke: temporal profile and outcome prediction

**DOI:** 10.1007/s00415-019-09477-9

**Published:** 2019-08-02

**Authors:** Annie Pedersen, Tara M. Stanne, Staffan Nilsson, Sofia Klasson, Lars Rosengren, Lukas Holmegaard, Katarina Jood, Kaj Blennow, Henrik Zetterberg, Christina Jern

**Affiliations:** 1 Department of Laboratory Medicine, Institute of Biomedicine, The Sahlgrenska Academy at University of Gothenburg, Box 440, 405 30 Gothenburg, Sweden; 2 Department of Clinical Genetics and Genomics, Sahlgrenska University Hospital, Gothenburg, Sweden; 3 Department of Mathematical Statistics, Chalmers University of Technology, Gothenburg, Sweden; 4 Department of Clinical Neuroscience, Institute of Neuroscience and Physiology, The Sahlgrenska Academy at University of Gothenburg, Gothenburg, Sweden; 5 Department of Psychiatry and Neurochemistry, Institute of Neuroscience and Physiology, The Sahlgrenska Academy at University of Gothenburg, Gothenburg, Sweden; 6 Clinical Neurochemistry Laboratory, Sahlgrenska University Hospital, Mölndal, Sweden; 7 Department of Neurodegenerative Disease, UCL Institute of Neurology, Queen Square, London, UK; 8 UK Dementia Research Institute at UCL, London, UK

**Keywords:** Biomarkers, Cerebrovascular disease, Prognosis, Stroke in young adults

## Abstract

**Background and purpose:**

Neurofilament light chain (NfL) is a marker of neuroaxonal damage. We aimed to study associations between serum NfL (sNfL) concentrations at different time points after ischemic stroke and outcomes.

**Methods:**

We prospectively included ischemic stroke cases (*n* = 595, mean age 59 years, 64% males) and assessed outcomes by both the modified Rankin Scale (mRS) and the NIH stroke scale (NIHSS) at 3 months and by mRS at 2 years. In a subsample, long-term (7-year) outcomes were also assessed by both mRS and NIHSS. We used the ultrasensitive single-molecule array assay to measure sNfL in the acute phase (range 1–14, median 4 days), after 3 months and 7 years in cases and once in controls (*n* = 595).

**Results:**

Acute-phase sNfL increased by the time to blood-draw and highest concentrations were observed at 3 months post-stroke. High sNfL associated to stroke severity and poor outcomes, and both associations were strongest for 3-month sNfL. After adjusting for age, previous stroke, stroke severity, and day of blood draw, 3-month sNfL was significantly associated to both outcomes at all time points (*p* < 0.01 throughout). For all main etiological subtypes, both acute phase and 3-month sNfL were significantly higher than in controls, but the dynamics of sNfL differed by stroke subtype.

**Conclusions:**

The results from this study inform on sNfL in ischemic stroke and subtypes over time, and show that sNfL predicts short- and long-term neurological and functional outcomes. Our findings suggest a potential utility of sNfL in ischemic stroke outcome prediction.

**Electronic supplementary material:**

The online version of this article (10.1007/s00415-019-09477-9) contains supplementary material, which is available to authorized users.

## Introduction

Serum neurofilament light chain (sNfL) has recently been suggested as a marker of neuroaxonal injury after ischemic stroke with potential applications both for patient monitoring and for observational and interventional studies [1]. NfL is a neuron-specific structural protein [2] and cerebrospinal fluid concentrations can be used as markers of neuronal damage [3]. The recent development of methods to quantify NfL in serum has led to several reports on sNfL in neurodegenerative disorders and traumatic brain injury [4–6] and opens the door for large studies on NfL as a marker of neuronal damage also in diseases where lumbar puncture is not part of the clinical routine, such as stroke. However, data on sNfL in ischemic stroke are still limited to a few published studies [1, 7–9]. Consistent with what would be expected of a marker of neuronal damage, results from these studies show higher sNfL in patients with acute ischemic stroke compared to both patients with a recent transient ischemic attack [8, 9] and controls [1, 7], and correlations between sNfL and infarct volume have been observed [1, 7]. There is evidence that sNfL increases with the time from symptom onset to blood-draw [1, 7, 8], and that sNfL remains elevated at 3 months post-stroke [1, 7]. Furthermore, recent data indicate that sNfL measured at day 7 after symptom onset, but not as early as within 24 h, is independently associated with 3-month functional outcome [1, 9], illustrating that the time point of measurement is of great importance when evaluating sNfL post-stroke.

Although reported data show promising results for sNfL as a biomarker in stroke, the post-stroke temporal profile and prognostic value remain to be evaluated in large samples also enabling analyses of relevant subgroups. In light of this, we aimed to explore sNfL in ischemic stroke in a longitudinal manner and to study associations for sNfL at different time points after ischemic stroke to both functional and neurological outcomes. We hypothesized that sNfL measured in the subacute or convalescent phase predicts post-stroke outcomes, and that the concentrations and/or the time profile of sNfL differ between etiologic subtypes.

## Materials and methods

### Study population

The study sample comprised of participants from the prospective Sahlgrenska Academy Study on Ischemic Stroke (SAHLSIS), which consecutively recruits patients with acute ischemic stroke aged 18–69 years at 4-stroke units, and has been described elsewhere [10]. The present study included cases recruited in a phase of the study when serum was biobanked (1998–2003, *n* = 600). Acute ischemic stroke was defined as an episode of focal neurological deficits with acute onset and lasting > 24 h and with no signs of hemorrhage on acute brain neuroimaging. All cases underwent computer tomography (CT) of the brain, and 65% also underwent magnetic resonance imaging (MRI) in the acute phase. Cases were subsequently excluded if further evaluation showed another etiology of the presenting symptoms than stroke.

We also included controls that had been randomly selected from population registers to match the cases with regards to age, sex and geographical residence area, as described [10]. Controls participated in a study visit, and only individuals with no history of stroke, coronary heart disease or peripheral arterial disease and/or signs of ischemic heart disease on resting electrocardiogram (ECG) were included (*n* = 600). Details on the selection of controls are described in the Supplemental Methods (online resource 1).

### Baseline characteristics, stroke severity, subtypes, and recurrent stroke

Information on cardiovascular risk factors, cardiovascular comorbidities, and recurrent strokes was collected as described in the Supplemental Methods (online resource 1). To obtain information on neurological comorbidities we used overlapping methods. First, we used information from a question on comorbidity that was included in a questionnaire distributed to all participants at baseline and to cases participating in the 7-year follow-up. Through this approach we identified one patient with self-reported multiple sclerosis and one with Parkinson’s disease. Additionally, we searched the Swedish Hospital Discharge register between 1998–2011, thus including the total follow-up period for all participants, for ICD-10 codes G10–G14, G20–G26, G30–G32, and G35–G37. Participants with a neurological disease with probable influence on sNfL measurement or outcome assessment (within 1 year from assessment) were identified as follows: 5 patients with the diagnoses G209, G255, G258, G300 or G328, and 2 controls with the diagnoses G209 or G258. Thus, in total seven patients and two controls were identified with a neurological comorbidity. Index stroke severity was scored as maximum severity within the first 7 days using the Scandianvian Stroke Scale (SSS). We converted the SSS to the more commonly used NIH stroke scale (NIHSS) using an algorithm [11]. Etiological subtypes were classified according to the Trial of Org 10172 in Acute Treatment (TOAST) criteria [12] with minor modifications as described [10]. According to this classification cryptogenic stroke is defined as a non-lacunar stroke with no identified etiology despite complete evaluation. Thus, this definition is similar to the definition of embolic stroke of undetermined source (ESUS) in recent randomized trials, for further information please see the Supplemental Methods (online resource 1). Patients were also subdivided according to the Oxfordshire Community Stroke Project (OCSP) classification [13].

### Outcomes at 3 months and 2 years

All cases were invited to a 3-month follow-up visit. A stroke neurologist (KJ) assessed functional outcome by the modified Rankin Scale (mRS) and scored neurological deficit (SSS converted to NIHSS as described above). After 2 years mRS was scored again through a telephone interview by a study nurse (IE) trained in stroke medicine and specifically trained to score the mRS by the same stroke neurologist who performed the 3-month follow-up.

### Substudy on long-term outcomes

Cases recruited at one of the stroke units, the main center at the Sahlgrenska University Hospital, who were alive at 7 years (*n* = 358 out of 411) were invited to a long-term follow-up visit, and 296 (83%) participated. Reasons for drop-outs have been described in detail elsewhere [14]. The same study nurse (IE) scored mRS and a stroke neurologist scored NIHSS. For 7-year mRS and NIHSS analyses, cases were limited to those with available 3-month sNfL measurements. For NIHSS, 272 individuals were included (*n* = 5 missing NIHSS scores and *n* = 19 missing 3-month sNfL data). Among surviving participants, data for analyzing mRS data were missing from 19 individuals (*n* = 3 missing mRS and *n* = 16 missing 3-month sNfL). For the analyses of mRS we also included individuals who had died during follow-up. Of the 53 individuals who had died, sNfL was available in 43, bringing the total substudy count for 7-year mRS to 320 individuals.

### Blood sampling and sNfL measurement

We collected blood samples, as described in the Supplemental Methods (online resource 1), from patients in the acute phase [median 4 days after index stroke, interquartile range (IQR) 3–6], at 3 months (median 101 days, IQR 94–111), and at 7 years (median 7.5 years, IQR 7.4–7.6) after index stroke. With regard to the acute phase, 80% of the cases were sampled within 6 days from stroke onset, and the numbers of cases that were sampled at different time points are displayed in Supplemental Fig. 1 (online resource 1). Controls were sampled once at inclusion. All samples were aliquoted and stored at − 80 °C pending analysis. sNfL concentrations were measured using a homebrew assay on the single-molecule array (SiMoA) platform (Quanterix, Lexington, MA) [15], as described [4]. The analyses were performed by board-certified laboratory technicians who were blind to clinical information, and all samples were analyzed on the same occasion with the same batch of reagents. The lower limit of quantification was 6.7 pg/mL. The intra-assay coefficient of variation was 6.4% for quality control (QC) samples with concentrations of 12.4 pg/mL and 110 pg/mL (no difference between the low and high QC sample in this study). The success rate for NfL measurements was > 99%.

### Statistical analyses

Functional outcome was assessed as a binary outcome (mRS score 0–2 vs 3–6), and the NIHSS score was treated as a numeric scale. For all statistical tests sNfL concentrations were log_10_ transformed. Baseline characteristics were compared using the *χ*^2^ test for proportions and Student’s *t* test or Mann–Whitney *U* test for numeric variables. Correlations were assessed by Pearson’s correlation coefficients. Associations between sNfL and day of blood sampling were analyzed by linear regression with stroke severity (baseline NIHSS) and age as covariates. Linear regression was used to analyze associations between sNfL and stroke severity and neurological outcome (NIHSS). Associations between sNfL and functional outcome (mRS) were analyzed by logistic regression. All regression analyses of associations to outcomes were performed univariably as well as adjusted for age, previous stroke (before index stroke) and stroke severity. Analyses including acute phase and 3-month sNfL were additionally adjusted for day of blood sampling during the acute phase and at 3-month follow-up, respectively. To assess the diagnostic accuracy of sNfL for discriminating good (mRS ≤ 2) and poor (mRS > 2) outcomes, we calculated the area under the receiver operating characteristics (ROC) curve.

sNfL in cases and controls were compared by Student’s paired *t* test and in multivariable analyses by mixed models using the paired sNfL as a repeated measure and including hypertension, diabetes mellitus, smoking, and hyperlipidemia as covariates. As sNfL increases with age and controls only were sampled at baseline, analyses of sNfL 7-year post-stroke in relation to controls were additionally adjusted for age. Differences in sNfL between the etiological subtypes were analyzed with ANCOVA adjusting for age. The decline in sNfL from 3 months to 7 years was compared between subtypes by ANOVA using age-adjusted concentrations for the measurement at 7 years and expressed as percent decline. Differences in sNfL according to OCSP subtypes were analyzed with ANOVA (post-hoc Tukey).

R 3.3.1 was used for analyzing ROC curves (package pROC). SPSS 20.0 was used for all other analyses. Two-tailed *p* < 0.05 was considered significant. More details can be found in the Supplemental Methods (online resource 1).

## Results

In five patients serum was unavailable from all time points, leading to a study cohort of 595 patients with serum sample available from at least one time point and their corresponding matched controls. Baseline characteristics and outcomes for the whole group and for the four main etiological stroke subtypes are shown in Table 1. As expected, the proportion of participants with cardiovascular risk factors and cardiovascular comorbidities was higher in cases than in controls. In cases, there were correlations between sNfL and stroke severity (*r* = 0.38 and *r* = 0.56 for acute phase and 3-month sNfL, respectively). sNfL was also correlated to age in controls (*r* = 0.50), whereas all other correlations between sNfL and baseline characteristics were weak, |*r*| ≤ 0.2. Baseline characteristics for the subgroup who participated in the 7-year follow-up are shown in Supplemental Table 1 (online resource 1).

**Table 1:** Baseline characteristics, sNfL concentrations, stroke severity (NIHSS baseline) and stroke outcomes (NIHSS and mRS) for the study participants

	Controls (*n* = 595)	Cases (*n* = 595)	Large vessel disease (*n* = 72)	Small vessel disease (*n* = 123)	Cardioembolic stroke (*n* = 97)	Cryptogenic stroke (*n* = 161)
Age, median (IQR)	59 (52–65)	59 (52–65)	60 (57–65)	60 (54–64)	61 (54–66)	56 (48–62)***
Male sex, *n* (%)	382 (64)	382 (64)	53 (74)	76 (62)	66 (68)	95 (59)
Hypertension, *n* (%)	220 (37)	350 (59)***	43 (60)***	88 (72)***	49 (51)**	87 (54)***
Diabetes mellitus, *n* (%)	33 (6)	112 (19)***	24 (33)***	26 (21)***	19 (20)***	22 (14)***
Hyperlipidemia, *n* (%)	398 (67)	413 (69)**	53 (74)*	77 (63)	73 (75)**	107 (66)
Current smoker, *n* (%)	108 (18)	230 (39)***	39 (54)***	53 (43)***	33 (34)***	59 (37)***
Previous history of stroke, *n* (%)	0 (0)	114 (19)***	21 (30)***	25 (20)***	22 (23)***	18 (11)***
History of coronary artery disease, *n* (%)	0 (0)	101 (17)***	16 (22)***	9 (7)***	39 (40)***	16 (9)***
Statins at 3 months, *n* (%)	31 (5)	198 (33)***	36 (50)***	40 (33)***	32 (33)***	55 (34)***
Anti-hypertensive drugs at 3 months, *n* (%)	87 (15)	290 (49)***	35 (49)***	70 (57)***	64 (66)***	61 (38)***
Anticoagulant drugs at 3 months, *n* (%)	0	111 (19)***	8 (11)***	2 (2)**	60 (62)***	17 (11)***
NIHSS score baseline, median (IQR)	NA	2.9 (1.6–7.2)	3.3 (1.2–11.5)	2.5 (1.6–4.2)	3.8 (1.2–11.1)	2.5 (1.2–6.5)
NIHSS score 3 months, median (IQR)	NA	0.4 (0.4–1.9)	1.2 (0.4–2.7)	0.4 (0.4–1.5)	0.8 (0.4–2.7)	0.4 (0.4–1.9)
NIHSS score 7 years, median (IQR)^a^	NA	0 (0–2)	1 (0–5)	0 (0–1)	0 (0–4)	0 (0–1)
mRS score 3 months, median (IQR)	NA	2 (1–2)	2 (1–3)	1 (1–2)	2 (1–3)	2 (1–2)
mRS score 2 years, median (IQR)	NA	2 (1–2)	2 (1–3)	1 (1–2)	2 (1–3)	2 (1–2)
mRS score 7 years, median (IQR)^a^	NA	2 (1–4)	3 (2–6)	2 (0–3)	3 (2–6)	2 (1–3)
sNfL acute (pg/mL), median (IQR)	14.2 (9.7–21)	60.2 (28.3–190.4)***	133.4 (54.7–395.5)***	33.3 (18–70.3)***	101.2 (43.4–324.3)***	51.6 (26.2–155.7)***
sNfL 3 months (pg/mL), median (IQR)	NA	90.6 (41.8–230.3)***	190 (70.3–336.7)***	55.6 (28.6–109.6)***	136 (59.6–390.9)***	81.9 (36.2–215.4)***
sNfL 7 years (pg/mL), median (IQR)^a^	NA	18.1 (11.5–35.5)***	22.0 (14.5–39.9)**	23.8 (13.1–39.6)**	21.0 (17.6–23.4)**	17.3 (11.3–28.9)***

Data are shown as median and interquartile range (IQR) or number (*n*) and percentage for controls and for the whole group of ischemic stroke cases as well as for the four major ischemic stroke subtypes. The remaining cases were of either other determined cause (*n* = 51) or undetermined cause (*n* = 91). Differences compared to the control group were examined using the *χ*^2^-test for proportions and Mann–Whitney *U* test for continuous variables, except for sNfL concentrations that were examined with paired *t* test on log-transformed sNfL values

*NIHSS* NIH stroke scale, *mRS* modified Rankin Scale, *sNfL* serum neurofilament light chain

*p < 0.05, **p < 0.01, ***p < 0.001

^a^Please note that the 7-year data are from a substudy including a subset of cases from the main study

### Serum NfL concentrations vary over time after acute ischemic stroke

**Fig. 1:**
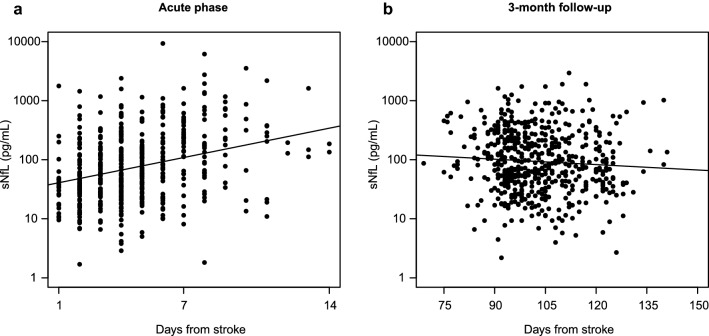
Correlations between sNfL and day of blood sampling during the acute phase (*r* = 0.32, *p* < 0.001, **a**) and at 3-month follow-up after ischemic stroke (*r* = −  0.08, *p* = 0.06, **b**). *sNfL* serum neurofilament light chain

Concentrations of sNfL were determined in 489 and 546 stroke cases in the acute phase and at 3 months, respectively. From 1998 to 2000, serum was not collected in the acute phase, explaining the majority (*n* = 78) of the missing values. Acute phase sNfL increased with time to blood-draw (*r* = 0.32), Fig. 1a. This association remained after adjustment for age and stroke severity in a linear regression model (*p* < 0.001). With regards to 3 months, sNfL decreased slightly with the time to blood sampling (*r* = − 0.08), Fig. 1b. This association was significant in a linear regression model adjusting for age and stroke severity (*p* < 0.01).

**Fig. 2:**
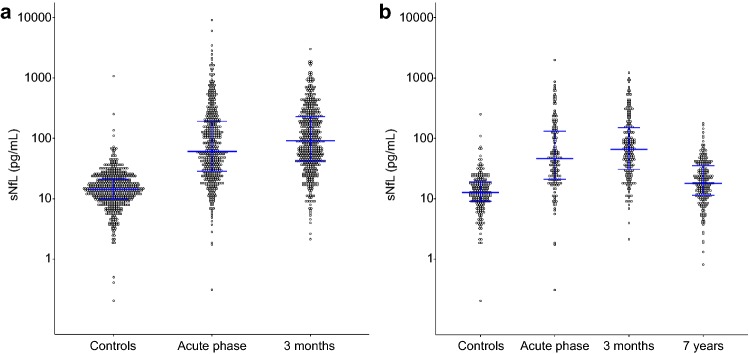
sNfL concentrations in ischemic stroke cases at different time points post-stroke and in controls. **a** sNfL concentrations in cases in the acute phase and at 3 months and in controls. **b** sNfL concentrations for the different time points for cases participating in the substudy on long-term outcomes (*n* = 221) and the matched controls (*n* = 221). Horizontal lines represent median concentrations and interquartile ranges. *sNfL* serum neurofilament light chain

Distributions of sNfL for the whole group are displayed in Fig. 2a. Acute phase and 3-month concentrations were correlated (*r* = 0.65), but 3-month sNfL was significantly higher than acute phase sNfL (*p* < 0.001). For all cases who had measurements of sNfL at both time points, the individual sNfL levels are displayed in Supplemental Fig. 2 (online resource 1), also illustrating the influence of the day of acute blood sampling. Although mean sNfL was highest at 3 months, the figure also shows that there was individual variability. In the group of cases with early blood sampling during the acute phase, a larger proportion had highest sNfL at 3 months compared to in the group with late acute sampling. Concentrations of sNfL from the 7-year follow-up visit were available for 221 individuals. Distributions of sNfL for these cases and their matched controls are displayed in Fig. 2b. Also in this subgroup we detected highest concentrations at 3 months post-stroke.

### Serum NfL is associated with stroke outcomes

Since our results indicate that the post-stroke peak sNfL concentrations in our study is best captured by the 3-month sample, we primarily assessed the association between 3-month sNfL and outcomes. High 3-month sNfL was strongly associated both with poor neurological outcome, i.e., high NIHSS score, at the 3-month follow-up and with poor functional outcome, i.e., mRS > 2, at both 3 months and 2 years (Table 2). In the substudy on long-term (7-year) outcomes, high sNfL was also significantly associated with both NIHSS and mRS (Table 3). All associations between 3-month sNfL and outcomes were independent of age, history of stroke, stroke severity and day of 3-month blood draw (Tables 2, 3). After exclusion of participants who experienced a recurrent stroke during follow-up, all associations where slightly attenuated, but retained (e.g., OR for poor functional outcome 19.0, 12.2 and 5.7 per log unit increase in sNfL at the 3-month, 2-year and 7-year follow-up, respectively, *p* < 0.001 for all three).

**Table 2:** Results from regression analyses showing associations to outcomes (NIHSS and mRS) at 3 months and 2 years

	NIHSS at 3 months		mRS > 2 at 3 months		mRS > 2 at 2 years	
	*β*	95% CI for *β*	OR	95% CI for OR	OR	95% CI for OR
Log sNfL 3 months	2.59	2.24–2.94	19.66	10.61–36.43	11.47	6.65–19.79
Log sNfL 3 months^a^	2.69	2.33–3.05	24.82	12.73–48.40	11.52	6.52–20.38
Log sNfL 3 months^b^	0.99	0.65–1.33	5.83	2.72–12.47	3.24	1.67–6.29
NIHSS score baseline	0.35	0.33–0.38	2.39	1.31–1.47	1.24	1.19–1.29
NIHSS score baseline^c^	0.35	0.33–0.38	1.39	1.32–1.47	1.26	1.21–1.31
NIHSS score baseline^d^	0.29	0.25–0.32	1.29	1.21–1.38	1.20	1.14–1.27

Multivariable linear regression models were used for calculation of *β*, i.e., change in NIHSS at 3 months, per log unit increase in sNfL (one log unit represents a tenfold increase). Multivariable logistic regression models were used for calculation of odds ratio per log unit increase in sNfL for poor outcome (mRS > 2) at 3 months and 2 years. For cases with 3-month sNfL measurements, 3-month NIHSS was available in 522 cases, 3-month mRS in 532 cases, and 2-year mRS in 542 cases

*sNfL* serum neurofilament light chain, *NIHSS* NIH stroke scale, *mRS* modified Rankin Scale

^a^Adjusted for age, history of stroke, and day for blood sampling at 3-month follow-up

^b^Adjusted for age, history of stroke, day for blood sampling at 3-month follow-up, and baseline NIHSS

^c^Adjusted for age and history of stroke

^d^Adjusted for age, history of stroke, 3-month log sNfL and day for blood sampling at 3-month follow-up

**Table 3:** Results from regression analyses showing associations to outcomes (NIHSS and mRS) in the substudy assessing 7-year outcomes

	NIHSS at 7 years		mRS > 2 at 7 years	
	*β*	95% CI for *β*	OR	95% CI for OR
Log sNfL 3 months	3.82	3.11–4.53	5.55	3.31–9.29
Log sNfL 3 months^a^	3.77	3.03–4.50	6.61	3.45–10.89
Log sNfL 3 months^b^	1.76	0.91–2.60	2.86	1.45–5.65
NIHSS score baseline	0.42	0.36–0.49	1.18	1.12–1.24
NIHSS score baseline^c^	0.42	0.36–0.49	1.20	1.14–1.27
NIHSS score baseline^d^	0.31	0.23–0.39	1.13	1.06–1.20

Multivariable linear regression models were used for calculation of *β*, i.e., change in NIHSS at 7 years, per log unit increase in sNfL (one log unit represents a tenfold increase). Multivariable logistic regression models were used for calculation of odds ratio per log unit increase in sNfL for poor outcome (mRS > 2) at 7 years. For 7-year mRS 3-month sNfL was available in 320 cases and for 7-year NIHSS in 272 cases

^a^Adjusted for age, history of stroke, and day for blood sampling at 3-month follow-up

^b^Adjusted for age, history of stroke, day for blood sampling at 3-month follow-up, and baseline NIHSS

^c^Adjusted for age and history of stroke

^d^Adjusted for age, history of stroke, 3-month log sNfL and day for blood sampling at 3-month follow-up

The associations between acute phase sNfL and outcomes were generally weaker than those for 3-month sNfL. These results are displayed in the Supplemental Tables 2 and 3 (online resource 1). The associations with 3-month functional outcome was similar for cases with early (≤ day 4) vs late (> day 4) blood sampling during the acute phase. For cases with very early blood sampling (day 1, *n* = 24) there was no significant association to 3-month mRS in univariable analysis. Since an association to 3-month mRS has been previously reported for sNfL at day 7 post-stroke [1], we also analyzed cases with acute blood sampling at day 6–8 (*n* = 129) separately. A univariable association was found, that was not retained after adjustment for age, previous history of stroke and stroke severity OR 2.2 (0.4–10.5).

Excluding patients with a neurological comorbidity yielded similar results for all outcome analyses (data not shown).

We next evaluated the diagnostic accuracy of sNfL for predicting functional outcome (mRS 0–2 vs 3–6) at 2 years post-stroke. Acute phase and 3-month sNfL yielded an AUC of 0.68 (95% CI 0.63–0.74) and 0.79 (95% CI 0.74–0.84), respectively. For comparison, the corresponding AUC for baseline stroke severity, a well-known strong predictor, was 0.84 (95% CI 0.80–0.88). Including age, sex, hypertension, diabetes mellitus, and smoking, in addition to baseline stroke severity, in the same model yielded an AUC of 0.86 (95% CI 0.82–0.89). When adding 3-month sNfL to the latter model, the AUC increased slightly, but not significantly to 0.87 (95% CI 0.84–0.90). The corresponding AUCs for 7-year mRS were for acute phase and 3-month sNfL 0.69 (95% CI 0.62–0.76) and 0.73 (95% CI 0.67–0.79), respectively; and for baseline stroke severity 0.76 (95% CI 0.70–0.81). For clinical variables and baseline stroke severity the AUC was 0.81 (95% CI 0.76–0.86), which was similar when adding 3-month sNfL to the model. Receiver operating characteristics curves for the 2-year and 7-year outcomes are displayed in Supplemental Figs. 3 and 4 (online resource 1). AUCs for acute phase sNfL and 3-month mRS are displayed in the Supplemental results (online resource 1).

### Serum NfL is higher in cases than in controls and differs by etiologic subtype

We also measured sNfL in all 595 controls, and sNfL was higher in cases compared to controls both in the acute phase and 3 months post-stroke (Table 1). After adjustment for cardiovascular risk factors, the difference between cases and controls was retained for both time points [the ratio of sNfL in cases vs controls was 5.0 (95% CI 4.4–5.9) for the acute phase and 6.5 (6.0–7.2) for 3-month concentrations]. Exclusion of participants who experienced a recurrent stroke within 3 months yielded similar results for 3-month sNfL [6.2 (5.4–6.9)]. After 7 years sNfL had declined, but was still significantly higher than in controls. However, this difference was not retained when adjusting for age and cardiovascular risk factors (ratio of sNfL in cases vs controls, 1.1, 95% CI 1.0–1.3). Excluding participants with neurological comorbidities yielded similar results as described above for all time points (data not shown).

**Fig. 3:**
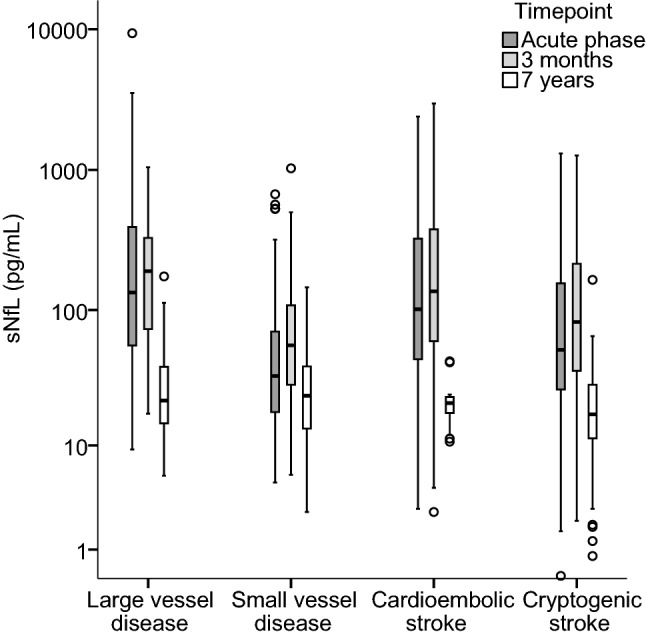
Distribution of sNfL concentrations in the acute phase, at 3 months and at 7 years in the four main etiologic subtypes of ischemic stroke. *sNfL* serum neurofilament light chain

sNfL in the four main etiologic subtypes is presented in Table 1, and the distributions of sNfL are displayed in Fig. 3. sNfL concentrations were significantly higher in each subtype compared to their matched controls. The highest acute phase and 3-month concentrations were observed in large vessel disease (LVD) and in cardioembolic (CE) stroke. At the 7-year follow-up the subtype pattern was different with highest sNfL in small vessel disease (SVD) (Fig. 3), but after adjustment for age this difference was not retained. The decline over time from 3 months to 7 years differed significantly between SVD (63%) and each of LVD (85%), CE (84%) and cryptogenic stroke (80%) (*p* < 0.05, throughout).

### Serum NfL in relation to OCSP classification

Three-month sNfL differed significantly between each pair of the subtypes total anterior circulation infarct (TACI), partial anterior circulation infarct (PACI), posterior circulation infarct (POCI) and lacunar infarct (LACI). The same was true for the acute phase concentrations except that there was no significant difference between PACI and POCI (*p* = 0.99). In the acute phase, the LACI group had lowest sNfL, followed by POCI, PACI, and TACI groups in the order of increasing sNfL.

## Discussion

In this cohort of ischemic stroke, circulating sNfL concentrations increased with the day of blood draw in the acute phase after index stroke, were highest at 3 months, and had declined 7 years post-stroke. sNfL at 3 months post-stroke showed strongest independent associations to both short- and long-term outcomes. Finally, we observed that the dynamics of sNfL differ by subtype of ischemic stroke.

For the first time we show that sNfL predicts both functional and neurological outcomes both in the short- and long-term after overall ischemic stroke. In our cohort, associations between acute phase and 3-month sNfL and outcomes were independent of age and stroke severity, which is in line with a recent but smaller study (*n* = 110) with data on sNfL measured at day 7 from admission [1]. In contrast, no independent association between sNfL and 3-month mRS was detected when blood was drawn within 24 h from symptom onset [9]. Thus, according to the present and previous studies [7, 8], 24 h is too early to catch the sNfL peak. In a study on non-traumatic cervical artery dissection [8], blood was drawn at similar time points as the acute phase sampling in the present study, but an association between sNfL and 3-month mRS did not remain after adjustment for stroke severity [8], which could be due to the small sample size (*n* = 49) and the fact that the electrochemiluminescence immunoassay was used to measure sNfL, which is less sensitive compared to the method we used here [16]. We used the ultrasensitive SiMoA technology to measure sNfL levels longitudinally after stroke, and for the first time these were investigated in relation to outcomes measured by both NIHSS and mRS scores at different time points. These results need replication, but indicate that sNfL is a promising candidate for prediction of short- and long-term outcomes after ischemic stroke. In contrast to most previously studied biomarkers of stroke outcomes that have mainly focused on inflammatory and hormonal pathways, the neurofilament proteins are exclusively expressed in neurons and thereby represent markers specific for neuroaxonal injury. This means they are independent of the underlying causal pathways and provide a measure of brain injury with the advantage of being much more readily available compared to brain imaging techniques. However, as indicated above the time point of measurement seems to be crucial when evaluating sNfL post-stroke. The warranted next step is to establish the post-stroke temporal profile of sNfL. As discussed below, the peak is probably not fully captured in our or previous studies, and speculatively measurements of peak levels could add predictive ability of sNfL. However, repeated measurements might be even more valuable, by measuring the rates of increase and decrease in sNfL and, thus capturing the dynamics of post-stroke injury processes.

We observed significantly higher sNfL in ischemic stroke cases compared to controls in the acute phase, after 3 months and 7 years, and this was also true when excluding participants with neurological comorbidities. After 7 years the sNfL had declined, and the association to case–control status was not independent of age and cardiovascular risk factors/comorbidities. The magnitude of the sNfL increment within 3 months post-stroke was associated to stroke severity, i.e., baseline NIHSS, which is in line with previous data [8, 9] and expected since both are measures related to the magnitude of brain injury. In line with this, sNfL differed between the OCSP subtypes with lowest acute phase levels in LACI. We did not have data on infarct size and localization, but speculate that sNfL and the NIHSS score represent overlapping but complementary measures of the magnitude and functional consequences of brain damage. Furthermore, our results indicate that sNfL continues to increase several days after stroke. Similar associations between time of blood draw and sNfL have been reported in lacunar stroke [7], ischemic stroke caused by cervical artery dissection [8], and traumatic spinal cord injury [17]. Moreover, a recent study on a subgroup of patients with acute ischemic stroke (*n* = 89) who had measurements of sNfL on day 1, 2, 3, and 7 after hospital admission observed highest concentrations at day 7 [1] and a study on phosphorylated neurofilament heavy protein observed highest concentrations at 3 weeks post-stroke [18]. Interestingly, a small recent study on 30 ischemic stroke patients with repeated measurements of sNfL on day 0–1, 2–3, 7–9, 3 weeks and 3–5 months confirmed these findings with increasing sNfL during the acute phase and highest concentrations at 3 weeks [19]. In our study, sNfL 3 months post-stroke was significantly higher compared to the acute phase, but contrary to the acute phase, the 3-months concentrations were decreasing with the day of blood-draw. In addition, as depicted in Supplemental Fig. 1 (online resource 1), a larger proportion of patients with acute phase blood sampling ≤ 4 days post-stroke had higher sNfL at 3 months compared to patients with acute blood sampling later than 4 days post-stroke. Taken together, these data point towards a peak in sNfL somewhere between the acute phase and 3 months post-stroke. The half-life of NfL in humans is not known, but data from mice have suggested a half-life around 3 weeks [20]. Neurofilaments are abundant in large myelinated axons susceptible to Wallerian post-stroke degeneration and the time profile of immunohistochemical as well as radiological findings after stroke show a delayed and longstanding degenerative process fitting the release pattern of sNfL in this study [21, 22]. Thus, the prolonged release of NfL possibly reflects these processes. This is further supported by recent data showing a correlation between sNfL measured 6 months post-stroke and a quantitative measure of secondary neurodegeneration obtained from diffusion tensor imaging MRI [1]. Although we do not know when sNfL is peaking after stroke, it is reasonable to assume that this time point may vary with infarct size. In support of such a hypothesis, in contrast to our results on all ischemic stroke and previous on lacunar stroke [7], sNfL in amateur boxers had decreased after a 3-month period of rest from boxing [5], which could be due to a milder brain injury compared to stroke. Taken together, present data on the temporal profile of sNfL post-stroke are not sufficient to identify the post-stroke peak. Therefore, we cannot draw any conclusions on the optimal time(s) for measurement, but clearly the very first days after stroke are too early.

We found that the sNfL profile differs by etiologic subtype of ischemic stroke. LVD and CE stroke showed highest acute phase and 3-month concentrations, which is expected since those etiologies in general cause larger strokes. The median 3-month sNfL concentrations for LVD were 190 pg/mL, CE stroke 136 pg/mL and SVD 56 pg/mL. For comparison, reported sNfL concentrations were approximately 30 pg/mL in multiple sclerosis [6], 80 pg/mL in frontotemporal dementia [4], and 22 pg/mL in mild traumatic brain injury (amateur boxers sampled 7–10 days after boxing) [5]. It should be noted, however, that these comparisons are only indicative; the assays used in these studies are similar (the same antibody pair to measure NfL was used) but have not been formally standardized to a reference material or harmonized to each other. Given the promising results on NfL as a blood biomarker for neuronal injury, such standardization projects are highly warranted. At 7 years, SVD showed highest sNfL, although at this time point the difference between the subtypes was not statistically significant in our sample. It is plausible that the concentrations are constantly elevated in some patients with SVD, due to a progressive disease course. In line with this, previous studies on NfL in cerebrospinal fluid have demonstrated increased concentrations with increasing severity of white-matter lesions on magnetic resonance imaging (MRI) [23, 24]. Moreover, a study on patients with small subcortical infarcts (< 25 mm) showed elevated sNfL in patients with new, but clinically silent cerebral small vessel disease-related lesions on MRI at follow-up [7]. This finding was further supported by a recent study showing that ischemic stroke patients with recurrent ischemic lesions on MRI at 6 months had higher sNfL compared to those without new lesions [1].

Our study has several strengths. The longitudinal design with repeated blood-draws and assessments of outcomes in short- and long-term provide novel information on the dynamics of sNfL, its relation to ischemic stroke subtypes, and its potential as a predictor of ischemic stroke outcomes. Further, we assessed two different outcome measures. Functional outcome as assessed by the mRS represents a crude measure, but the dichotomization refers to differences of great importance clinically and for the patients. The NIHSS assesses neurological outcome and is more detailed compared to the mRS. Our study sample was large and comprised of consecutive, well-characterized and relatively young ischemic stroke patients, which is why the proportion of comorbidities was low. Blood sampling was strictly standardized and sNfL was measured by the most sensitive method available [16]. Our study also has limitations. Although we had serum samples available from three time points, and those in the first phase after stroke were spread over a period of time, we did not have repeated measurements during the acute phase and no measurements between the acute phase and 3 months. Therefore, our data are insufficient to identify the post-stroke peak in sNfL. Blood sampling in narrower time intervals over the first weeks and months is needed in future studies to determine the trajectories of sNfL after stroke. Moreover, index stroke severity was scored as maximum severity within the first 7 days and it is possible that some cases, especially those with early blood sampling, have experienced a deterioration leading to falsely low sNfL concentrations in relation to stroke severity. Moreover, sNfL is known to associate with cerebral small vessel burden [25]. Since we did not have available data on small disease burden for all cases or any of the controls, we were not able to account for this in our analyses. There were also several years between blood sampling and sNfL analyses. However, all samples were stored at − 80 °C pending analysis, and NfL is known to be stable at − 80 °C and has also been shown to be stable over multiple freeze–thaw cycles [26]. Finally, our study cohort consisted of young and middle-aged ischemic stroke patients which may reduce the external validity of our results.

## Conclusions

Patients with acute ischemic stroke have increased sNfL concentrations, and the dynamics of sNfL differ by stroke subtype. There is an association between sNfL and both neurological and functional outcomes, which is independent of stroke severity. The results from this study, which represent the largest analyses of sNfL after ischemic stroke and also include repeated measures of both sNfL and outcomes, improve our understanding of sNfL in ischemic stroke. Future studies should aim to establish the time profile of sNfL post-stroke to enable identification of the optimal time for measurements.

## Electronic supplementary material

Below is the link to the electronic supplementary material.

Supplementary file1 (DOCX 166 kb)
