# Supplementary material for: Circulating neurofilament light in ischemic stroke: temporal profile and outcome prediction
Source: J Neurol. 2019 Aug 2;266(11):2796–806. doi: 10.1007/s00415-019-09477-9 (PMC6803587; doi:10.1007/s00415-019-09477-9)
Supplement: Supplementary file 1 — Supplementary file1 (DOCX 166 kb) [file 415_2019_9477_MOESM1_ESM.docx]

**SUPPLEMENTAL MATERIAL**

*Journal of Neurology*

**Circulating neurofilament light in ischemic stroke: Temporal profile and outcome prediction**

Annie Pedersen, MD^1,2^, Tara M Stanne, PhD^1^, Staffan Nilsson, PhD^1,3,^, Sofia Klasson, MSc^1^, Lars Rosengren, MD, PhD^4^, Lukas Holmegaard, MD^4^, Katarina Jood, MD, PhD^4^, Kaj Blennow, MD, PhD^5,6^, Henrik Zetterberg, MD, PhD^5,6,7,8^, Christina Jern, MD, PhD^1,2^,

^1^Institute of Biomedicine, Department of Laboratory Medicine, the Sahlgrenska Academy at University of Gothenburg, Sweden

^2^Department of Clinical Genetics and Genomics, Sahlgrenska University Hospital, Gothenburg, Sweden

^3^Department of Mathematical Statistics, Chalmers University of Technology, Gothenburg, Sweden

^4^Institute of Neuroscience and Physiology, Department of Clinical Neuroscience, the Sahlgrenska Academy at University of Gothenburg, Sweden

^5^Institute of Neuroscience and Physiology, Department of Psychiatry and Neurochemistry, the Sahlgrenska Academy at University of Gothenburg, Sweden

^6^Clinical Neurochemistry Laboratory, Sahlgrenska University Hospital, Mölndal, Sweden

^7^Department of Molecular Neuroscience, UCL Institute of Neurology, Queen Square, London, United Kingdom

^8^UK Dementia Research Institute at UCL, London, United Kingdom

**Corresponding author:** Annie Pedersen. E-mail: [annie.pedersen@gu.se](mailto:annie.pedersen@gu.se)

**Supplemental Methods**

*Selection of controls, data on recurrent strokes and neurological comorbidities*

Controls were randomly selected from participants in a population-based health survey [1] or the Swedish Population Register to match cases with regards to age, sex and geographical residence area. In total 1,107 controls were contacted. Of those, 208 did not respond, and 191 were unwilling to participate. 108 fulfilled the exclusion criteria of either a history of stroke, coronary heart disease or peripheral arterial disease and/or signs of ischemic heart disease on electrocardiogram (ECG).

For all participants we collected data on recurrent strokes for the whole follow-up period. We used questionnaires and the National Hospital Discharge Registry. We confirmed all events by reviewing the corresponding medical record, according to criteria as previously described [2]. To ensure that all early recurrences were identified, the stroke neurologist also sought recurrent events by medical history and review of medical records at the three-month follow-up visit.

*Etiologic subtypes and baseline data*

Of note, our definition of cryptogenic stroke does not include cases with incomplete evalution, but only those for whom the etiology was not found despite an extensive work-up [3]. Cases underwent work-up according to clinical routine. All cases underwent cardiac rhythm monitoring or repeated ECGs, and 77% underwent TTE and/or TEE. Extracranial cerebral arteries were visualized by Doppler ultrasound (in 82%) and intracranial cerebral arteries (in 32%) by MRA, CTA and/or catheter angiography. As the definition of embolic stroke of undetermined source (ESUS) in the recent randomized trials did not require visualization of the intracranial arteries, the group of patients defined as cryptogen stroke is similar to the definition of ESUS in these trials [4, 5]. Information on vascular risk factors was collected at inclusion for controls, and at inclusion and at a three-month follow-up for cases, by examinations and a structured questionnaire, as described [3]. Hypertension was defined as pharmacological treatment for hypertension and/or systolic blood pressure ≥160 mm Hg, and/or diastolic blood pressure ≥90 mm Hg. Diabetes mellitus was defined as dietary or pharmacological treatment and/or fasting plasma glucose ≥7.0 mmol/L. Hyperlipidemia was defined by pharmacological treatment, total fasting serum cholesterol >5.0 mmol/L, and/or low-density lipoprotein >3.0 mmol/L. Among cases measurements performed at 3-month follow-up were used to define hypertension, diabetes, and hyperlipidemia. Smoking was coded as current versus never or former (smoking cessation at least one year from inclusion). History of coronary artery disease was defined as having suffered myocardial infarction or having ECG changes indicating previous myocardial infarction.

*Blood sampling*

The number of cases that were sampled at different time-points during the acute phase are illustrated in Supplemental Figure I. Blood was collected between 8.30 and 10.30 a.m. after an overnight fast. Serum was isolated within 2 h by centrifugation 2000 x *g* at 4 °C for 20 min.

*Statistical analyses*

Missing values for the covariates (hypertension, n=9; smoking, n=3; diabetes n=2, hyperlipidemia n=50) were replaced by dummy variables in the multivariable analyses.

Based on their known or plausible influence on sNfL concentrations and post-stroke outcomes we selected the variables age [6], previous stroke (before index stroke), and stroke severity (baseline NIHSS) for the multivariable models assessing outcomes. In addition, day of blood sampling during the acute phase and at 3-month follow-up was added for analyzes of acute and 3-month sNfL, respectively. Etiologic subtype was considered as a covariate, but after inclusion of stroke severity it was no longer significant and was therefore deselected. In individuals with available sNfL concentrations there were no missing values for age, previous stroke, baseline NIHSS or etiologic subtype. Sensitivity analyses excluding participants with neurological comorbidities were performed for analyses of sNfL in cases vs controls and for analyses of post-stroke outcomes. For outcome analyses we additionally performed sensitivity analyses excluding participants who experienced a recurrent stroke during follow-up.

**Supplemental Results**

In cases, acute phase sNfL concentrations were significantly correlated to baseline stroke severity, age and endovascular treatment. Only five patients received endovascular treatment and they also had significantly higher NIHSS scores compared to the whole sample as well as relatively late blood sampling during the acute phase (day 2, 7, 8, 8 and 10).

We evaluated the diagnostic accuracy of acute phase sNfL for predicting functional outcome (mRS 0-2 vs 3-6) at three months by assessing the AUC. Acute phase sNfL and days to blood sampling during the acute phase in the same model yielded an AUC of 0.73 (95% CI, 0.67-0.79). A model with age, sex, hypertension, diabetes, smoking, and stroke severity (baseline NIHSS score) yielded an AUC of 0.91 (95% CI, 0.88-0.94). Adding acute phase sNfL and days to blood sampling during the acute phase to the latter model only marginally improved the diagnostic accuracy to 0.92 (95% CI, 0.89-0.95).

**Supplemental Tables**

- **Table 1** Baseline characteristics for the substudy of 7-year outcomes
- **Table 2** Associations between acute phase sNfL and NIHSS at 3 months
- **Table 3** Associations between acute phase sNfL and mRS at 3 months and 2 years

**Table 1.** Baseline characteristics, stroke severity (NIHSS score at baseline) and outcomes (NIHSS score and mRS at 3 months, 2 years and 7 years) for the participants in the substudy on 7-year outcomes after ischemic stroke.

|  | Substudy on 7-year outcome |
| --- | --- |
|  | (n = 320) |
| Age, median (IQR) | 57 (50 - 63) |
| Male sex, n (%) | 238 (64) |
| Hypertension, n (%) | 200 (54) |
| Diabetes mellitus, n (%) | 69 (19) |
| Hyperlipidemia, n (%) | 269 (73) |
| Current smoker, n (%) | 143 (39) |
| Previous history of stroke, n (%) | 70 (19) |
| History of coronary artery disease, n (%) | 61 (16) |
| NIHSS score baseline, median (IQR) | 2.9 (1.2 - 6.8) |
| NIHSS score 3 months, median (IQR) | 0.4 (0.4 - 1.9) |
| NIHSS score 7 years, median (IQR) | 0 (0 - 2) |
| mRS 3 months, median (IQR) | 2 (1 - 2) |
| mRS 2 years, median (IQR) | 2 (1 - 2) |
| mRS 7 years, median (IQR) | 2 (1 - 3) |

Data are shown as median and interquartile range (IQR) or number (n) and percentage. Please note that this table includes cases that were included in the analyses of 7-year outcomes, i.e. with available 3-month sNfL measurements as well as 7-year outcome data. NIHSS score, NIH stroke scale score; mRS, modified Rankin Scale.

**Table 2.** Linear regression analyses showing associations between acute phase sNfL (median day 4 post-stroke) and outcomes measured as NIHSS score at 3 months after index ischemic stroke.

|  | NIHSS 3 months | | |
| --- | --- | --- | --- |
|  | β | 95 % CI for β | p value |
| Log sNfL acute phase | 1.38 | 1.03-1.74 | < 0.001 |
| Log sNfL acute phase^a^ | 1.62 | 1.24-1.99 | < 0.001 |
| Log sNfL acute phase^b^ | 1.60 | 1.22-1.98 | < 0.001 |
| Log sNfL acute phase^c^ | 0.38 | 0.07-0.68 | 0.02 |

Multivariable linear regression models were used for calculation of β, i.e. change in NIHSS score at 3 months, per log unit increase in acute phase sNfL (one log unit represents a ten-fold increase). ^a^adjusted for day of blood sampling during the acute phase. **^b^**adjusted for age, history of stroke before index stroke and day of blood sampling during the acute phase. ^c^adjusted for age, history of stroke before index stroke, stroke severity (baseline NIHSS) and day of blood sampling during the acute phase.

sNfL; serum neurofilament light chain, NIHSS; NIH stroke scale

**Table 3.** Logistic regression analyses showing the odds ratios for poor functional outcome (mRS >2) at 3 months and 2 years after index ischemic stroke per log unit increase in acute phase sNfL.

|  | mRS >2 at 3 months | | | mRS >2 at 2 years | | |
| --- | --- | --- | --- | --- | --- | --- |
|  | OR | 95 % CI for OR | p value | OR | 95 % CI for OR | p value |
| Log sNfL acute phase | 4.26 | 2.75-6.60 | < 0.001 | 3.28 | 2.20-4.88 | < 0.001 |
| Log sNfL acute phase^a^ | 5.81 | 3.50-9.65 | < 0.001 | 3.89 | 2.50-6.07 | < 0.001 |
| Log sNfL acute phase^b^ | 5.86 | 3.51-9.78 | < 0.001 | 3.95 | 2.49-6.26 | < 0.001 |
| Log sNfL acute phase^c^ | 2.15 | 1.09-4.24 | 0.03 | 1.59 | 0.92-2.75 | 0.10 |

Multivariable logistic regression models were used for calculation of odds ratio per log unit increase in acute phase sNfL for mRS >2 at 3 months and 2 years, respectivley (one log unit represents a ten-fold increase).

^a^adjusted for day of blood sampling during the acute phase.

^b^adjusted for age, history of stroke before index stroke and day of blood sampling during the acute phase.

^c^adjusted for age, history of stroke before index stroke, stroke severity (baseline NIHSS) and day of blood sampling during the acute phase.

sNfL; serum neurofilament light chain, NIHSS; NIH stroke scale, mRS; modified Rankin Scale

**Supplemental Figures**

- **Fig. 1** The distribution of ischemic stroke cases that were sampled on different days during the acute phase.

**Fig. 2** Individual acute phase and 3-month sNfL values.

- **Fig. 3** ROC curves for good vs poor outcome (mRS 0-2 vs 3-6) at 2 years post-stroke.
- **Fig. 4** ROC curves for good vs poor outcome (mRS 0-2 vs 3-6) at 7 years post-stroke.

**Fig. 1** The distribution of number of ischemic stroke cases according to the day of blood draw in relation to stroke onset.


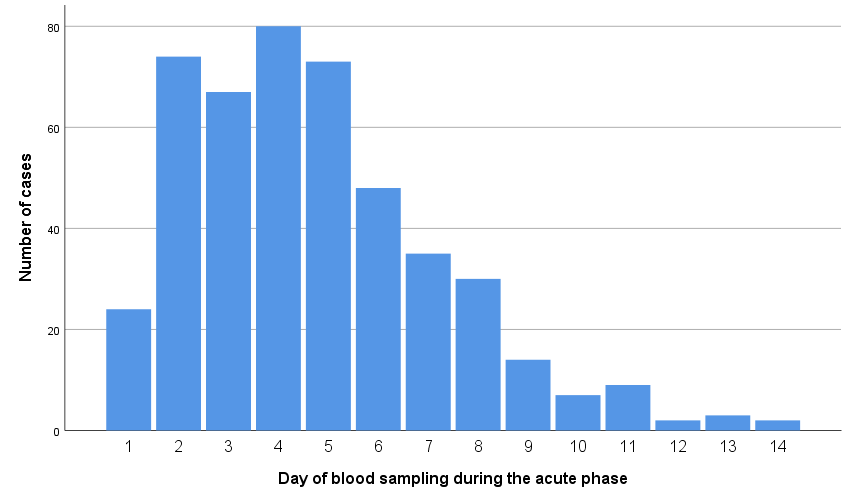


**Fig. 2** Individual acute phase and 3-month sNfL values in cases with ischemic stroke.

**
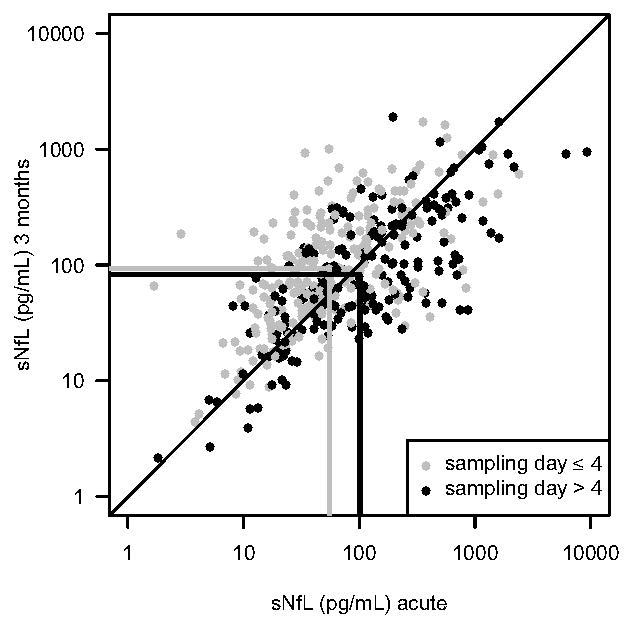
**

Early (within 4 days) vs late blood sampling during the acute phase displayed by grey and black symbols, respectively. The vertical and horizontal lines correspond to the geometric means.

sNfL, serum neurofilament light chain

**Fig. 3** ROC curves for good vs poor outcome (mRS 0-2 vs 3-6) at 2 years post-stroke.


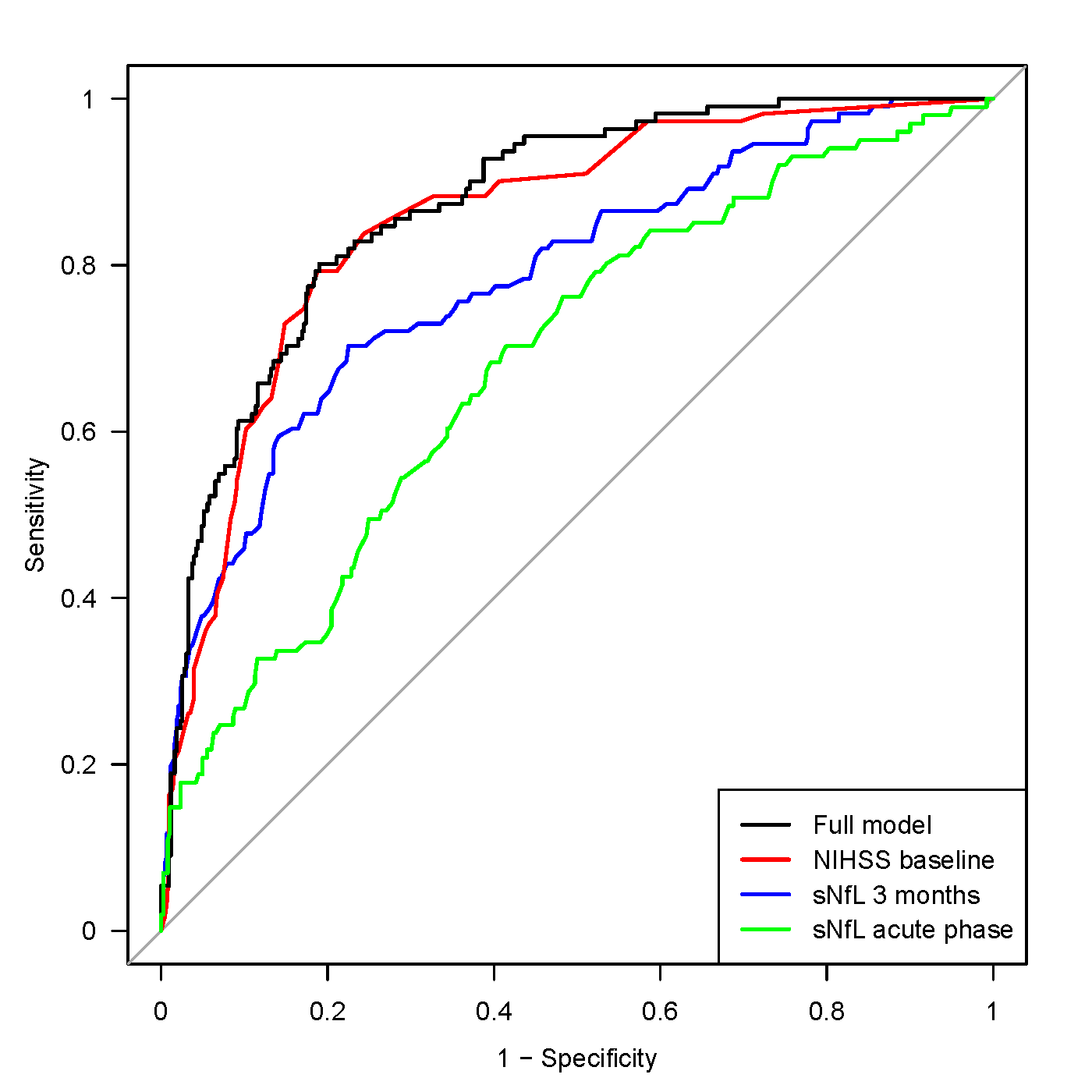


ROC curves for acute phase sNfL, 3-month sNfL, baseline NIHSS (stroke severity), and a full model including age, sex, hypertension, diabetes mellitus, smoking, baseline NIHSS and 3 months sNfL. ROC, Receiver operating characteristics; mRS, modified Rankin Scale; sNfL, serum neurofilament light chain; NIHSS, NIH stroke scale

**Fig. 4** ROC curves for good vs poor outcome (mRS 0-2 vs 3-6) at 7 years post-stroke.


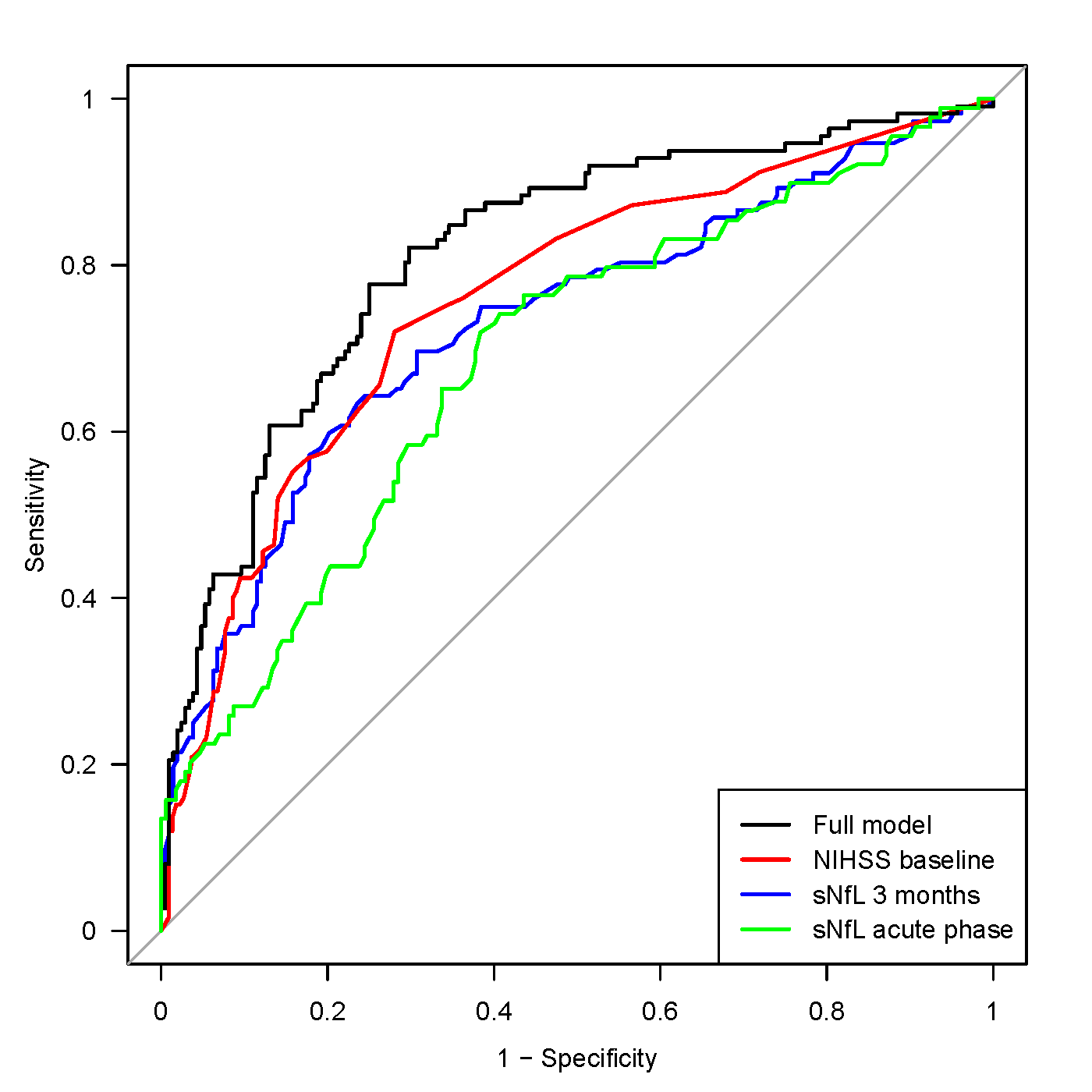


ROC curves for acute phase sNfL, 3-month sNfL, baseline NIHSS (stroke severity), and a full model including age, sex, hypertension, diabetes mellitus, smoking, baseline NIHSS and 3 months sNfL. ROC, Receiver operating characteristics; mRS, modified Rankin Scale; sNfL, serum neurofilament light chain; NIHSS, NIH stroke scale

**Supplemental References**

1. Wilhelmsen L, Johansson S, Rosengren A, Wallin I, Dotevall A, Lappas G (1997) Risk factors for cardiovascular disease during the period 1985-1995 in Goteborg, Sweden. The GOT-MONICA Project. Journal of internal medicine 242:199-211

2. Redfors P, Jood K, Holmegaard L, Rosengren A, Blomstrand C, Jern C (2012) Stroke subtype predicts outcome in young and middle-aged stroke sufferers. Acta neurologica Scandinavica 126:329-335

3. Jood K, Ladenvall C, Rosengren A, Blomstrand C, Jern C (2005) Family history in ischemic stroke before 70 years of age: The Sahlgrenska academy study on ischemic stroke. Stroke 36:1383-1387

4. Hart RG, Sharma M, Mundl H, Kasner SE, Bangdiwala SI, Berkowitz SD, Swaminathan B, Lavados P, Wang Y, Wang Y, Davalos A, Shamalov N, Mikulik R, Cunha L, Lindgren A, Arauz A, Lang W, Czlonkowska A, Eckstein J, Gagliardi RJ, Amarenco P, Ameriso SF, Tatlisumak T, Veltkamp R, Hankey GJ, Toni D, Bereczki D, Uchiyama S, Ntaios G, Yoon BW, Brouns R, Endres M, Muir KW, Bornstein N, Ozturk S, O'Donnell MJ, De Vries Basson MM, Pare G, Pater C, Kirsch B, Sheridan P, Peters G, Weitz JI, Peacock WF, Shoamanesh A, Benavente OR, Joyner C, Themeles E, Connolly SJ (2018) Rivaroxaban for Stroke Prevention after Embolic Stroke of Undetermined Source. The New England journal of medicine 378:2191-2201

5. Diener HC, Sacco RL, Easton JD, Granger CB, Bernstein RA, Uchiyama S, Kreuzer J, Cronin L, Cotton D, Grauer C, Brueckmann M, Chernyatina M, Donnan G, Ferro JM, Grond M, Kallmunzer B, Krupinski J, Lee BC, Lemmens R, Masjuan J, Odinak M, Saver JL, Schellinger PD, Toni D, Toyoda K (2019) Dabigatran for Prevention of Stroke after Embolic Stroke of Undetermined Source. The New England journal of medicine 380:1906-1917

6. Khalil M, Teunissen CE, Otto M, Piehl F, Sormani MP, Gattringer T, Barro C, Kappos L, Comabella M, Fazekas F, Petzold A, Blennow K, Zetterberg H, Kuhle J (2018) Neurofilaments as biomarkers in neurological disorders. Nature reviews Neurology 14:577-589
